# The incidence, prevalence, and contributing factors of overweight and obesity among adolescent population of India: A scoping review protocol

**DOI:** 10.1371/journal.pone.0275172

**Published:** 2022-09-26

**Authors:** Lopamudra Jena Samanta, Jayashree Parida, Jagatdarshi Badamali, Abinash Pradhan, Prasant Kumar Singh, Bijaya Kumar Mishra, Prasanna Kumar Patra, Sanghamitra Pati, Harpreet Kaur, Subhendu Kumar Acharya

**Affiliations:** 1 ICMR-Regional Medical Research Center, Bhubaneswar, Odisha, India; 2 ICMR- National Institute of Cancer Prevention and Research, New Delhi, India; 3 Department of Anthropology, Utkal University, Bhubaneswar, Odisha, India; 4 Division of ICMR, Division of Epidemiology and Communicable Diseases (ECD-Tribal Health), New Delhi, India; Kasturba Medical College, Manipal Academy of Higher Education, Manipal, INDIA

## Abstract

**Introduction:**

Overweight and obesity among the adolescent population are emerging as worldwide epidemics. Its increasing occurrence in India is highly concerning. Amidst the burden of malnutrition, where undernutrition is a long-standing health problem, the rising concerns around childhood overweight/obesity has several repercussions for this population. The aim of this scoping review is to map the evidence of the prevalence and contributing factors of overweight and obesity among adolescents (10 to 19 years) in the Indian population.

**Methods:**

The study will be conducted according to the Arksey and O’Malley scoping review framework and the Joanna Briggs institute Reviewers’ manual. The Population, Concept and Context strategy (PCC) will ensure the review questions, eligibility criteria, and search strategy. The Systematic Review and Meta-analysis: Extension for Scoping Review (PRISMA-ScR) will be used for the findings of the study of Scoping Review. The Mixed Methods Appraisal Tool (MMAT), Version- 2018 will be used to assess the methodological quality of all relevant studies. Literature search will be done using electronic databases: PubMed, Google Scholar, SCOPUS, EMBASE, and Cochrane library by specific keywords such as “prevalence”; “overweight”; “obesity”; “obese”; “malnutrition”; “BMI”; “adolescent”; “teenager”; and “youth” etc. Additional studies will be considered using cross-references.

## Introduction

Adolescence ranging from the age of 10–19 years is a transitional phase that leads to attaining puberty and adulthood. This phase involves major physical, psychological and behavioral changes and any adversity in this developmental phase may bring serious health outcomes involving chronic morbidity and mortality risks [1]. The major health concerns that emerge during the adolescence include malnutrition (both undernutrition and overweight), mental health problems, early pregnancy, and childbirth, human immune deficiency virus/ sexually transmitted infection (HIV/STI) and other infectious diseases, interpersonal violence, unintentional injuries, and substance abuse [2, 3]. It has been evident that 30–60% of causes of chronic diseases in adulthood are caused due to adverse health behavior during adolescence [4]. The global adolescent population is more than 1.2 billion. The vulnerability of adolescent age groups towards lifestyle diseases and the emerging trend of overweight and obesity among children and adolescents is a public health challenge worldwide [5]. According to the World Health Organization (WHO) 2016 report, the global prevalence rate of overweight among adolescents aged 10–19 years was over one in every six adolescents [6]. With 253 million adolescents (21% of the total population), India has the largest adolescent population in the world [3]. Several studies on malnutrition among Indian adolescents and on overnutrition, in particular, have reported the concerning state of rising overweight/obesity among adolescents [7, 8]. A 2019 study from South Indian Karnataka state revealed the prevalence of overweight and obesity at 10.8% and 6.2% respectively with a trend of higher prevalence among males than females [9]. Findings from Punjab in north part of the country highlighted the prevalence of obesity in rural and urban schools at 2.7% and 11% respectively along with the high prevalence of obesity-related hypertension among urban school children [10]. A similar finding was reported from western Gujarat state, where the prevalence of obesity and overweight among urban school-going children aged 10–18 years was higher to rural [11]. Adolescents from high-income families (OR = 2.35 (1.43–3.85)) as well as those who have a family history of obesity (OR = 2.4 (1.72–3.33) were observed to be more overweight or obese [12]. So, such findings highlight the need for appropriate action around extensive research, intervention, and behavioral modification in the line of adolescent nutritional health. However, the foremost need is to generate and synthesize high-quality data and evidence.

It has been observed that inadequate diet or insufficient physical activity (or both) are major reasons for overweight and obesity among adolescents [13]. According to the World Health Organization [14] report, childhood obesity has a higher risk towards acquiring obesity during adulthood and other associated non-communicable diseases including cardiovascular diseases, type-II diabetes, musculoskeletal disorder, certain cancers (breast, liver, gallbladder, kidney, colon, endometrial, ovarian, and prostate) [14]. Physical activity leads to physical expenditure, energy balance, and weight control and reduces the risk of cardiovascular diseases.

Despite several studies reporting the prevalence of adolescent obesity and overweight in many of the Indian states, the national-level estimation on the prevalence of obesity and overweight among Indian adolescents is inadequate. Very few literature reviews have been undertaken that focus on the incidence, prevalence, and contributing factors of obesity and overweight among adolescents in India. This scoping review is aimed at generating and synthesizing high-quality data to identify the knowledge gaps, clarify the concepts and map the evidence available [15]. This study will investigate the prevalence of overweight and obesity among the adolescent population in India and identify the major contributing factors influencing this prevalence rate. The findings of this review will form the basis for future research that will assist in identifying appropriate management strategies to achieve adolescent nutritional health in the Indian population. No previously published scoping review on overweight/obesity among the adolescent population of India was registered on PROSPERO, Fig share, Open Science Framework.

## Objectives

The objectives of the present scoping review are:

- To map the evidence on the incidence and prevalence of overweight and obesity among the adolescent population in India.
- To identify the contributing factors related to adolescent overweight and obesity and the patterns and trends of availability of such associated research evidence in India.

## Methods and analysis

In the present review, we aim to synthesis the evidences around selected objectives defining the prevalence and influencing factors of adolescent overweight/obesity. Prior to defining the objectives and design of the study, we first checked for the availability and accessibility of any scoping review on overweight/obesity status among the adolescent population of India in online database and protocol registration website. We checked it on PROSPERO, Fig share, Open Science Framework; consequently, we found that there is no previously published scoping review on overweight/obesity among adolescent population of India, followed by which the present study was designed.

The scoping study is also considered as a useful approach for determining the need and value of a future study. The methods using in this scoping review will follow the five stages of the Arksey and O’Malley scoping review framework and the Joanna Briggs Institute Reviewers’ Manual [16, 17]. Following are the five listed steps:

1. Identifying the research questions,
2. Identifying relevant studies,
3. Study selection,
4. Charting the data;
5. Collating, summarizing, and reporting results

The research questions, search strategy and eligibility criteria will be conducted based on the Population, Concept, and Context (PCC) strategy. The Mixed Methods Appraisal Tool (MMAT), Version- 2018 will be used to appraise the quality of empirical studies including qualitative, quantitative, and mixed-method studies [18]. The Preferred Reporting Items for Systemic Review and Meta-analysis Scoping Reviews (PRISMA-ScR) will be used [19]. The PRISMA-ScR checklist is given in **S1 File**.

### Identifying the research questions

To map the evidence on the incidence, prevalence and contributing factors of overweight/obesity in Indian adolescents, the research questions should be broad. Two overarching questions are developed for this scoping review:

- What is the range of evidence around incidence and prevalence of overweight and obesity among the vulnerable adolescent population in India?
- What is the status of present literature defining the contributing factors affecting adolescent overweight and obesity among the adolescent population in India?

### Identifying relevant studies

JBI recommends defining the following elements: ‘population’, ‘concept’, and ‘context’ which will guide the eligibility criteria.

### Search strategy

The reviewers will collect relevant literature which will be identified from different online databases such as PubMed, EMBASE, SCOPUS, Web of Science, Cochrane database, Google Scholar, and Google. This scoping review literature search will be performed by using the various MeSH terms such as, “prevalence”; “overweight”; “obesity”; “obese”; “malnutrition”; “nutritional status”; “BMI”; “adolescents”; “teenager”; “youth”; and “India” etc. All the grey literature, Govt. reports, short communication, and editorial letters will be excluded from the review. The reference list of the articles will also be searched to find out relevant articles. The searching strategy to be used for PubMed online database is given in **S2 File** (Draft of Minimal Dataset).

### Eligibility criteria

#### Inclusion criteria

- Population: The adolescent group aged from 10–19 years, for both sexes.
- Concept: Both the indicators- overweight and obesity, will be used for adolescents in this scoping review.
- Context: India
- Review period: Studies conducted from 2000 to 2022.
- Measuring scale: Studies that measure adolescent overweight and obesity (estimated quantitatively using the WHO scale which is BMI for age).
- Only studies in the English language.
- Studies with empirical data on incidence, prevalence, contributing factors (risk factors etc.) such as prevalence studies, case controlled, cross-sectional studies.

#### Exclusion criteria

- Studies undertaken prior to the year 2000, or considering population other than India.
- Studies targeting individuals below 10 years and above 19 years.
- Studies that do not measure BMI
- Papers with no original empirical data e.g., descriptive papers, commentaries, editorials etc.
- Interventional studies targeting treatment which lead to specific conditions like after hormone therapy weight gain among transgender, long term imbalance of Thyroid hormone, polycystic ovary syndrome and pregnant women as these conditions do not conform to the primary nutrition associated causes of overweight and obesity.

### Study procedure and selection of the studies

With an aim to ensure the comprehensiveness of the study, appropriate screening and selection procedures will be followed for the articles. Titles and abstracts of the original articles will be screened to identify potentially relevant articles and duplicate articles will be removed from the study by the investigators (L.J. and J.B.) followed by cross check by the reviewers (J.P. and P. K.S.). The already defined inclusion and exclusion criteria will be followed to select the relevant literature. The records identified by the reviewers will be included in the full-text screen; the same eligibility criteria will be followed to screen the full-text articles. Any disagreement among the reviewers will be resolved by mutual discussion or by referring to a third reviewer (S.K.A.). All the stages for selection of the relevant studies will be presented in a Preferred Reporting Items for Systemic Review and Meta-analysis Scoping Reviews (PRISMA-ScR) flow diagram.

### Charting the data

Three reviewers (L.J., J.B. and J.P.) will extract the relevant data from the selected literature. A predefined data extraction form will be developed using the MS-Excel spreadsheet. A summary table will be presented consisting of the key information of the selected studies. This information will include author’s information, year of the study, geographical regions, population age range, type of study, sexes, sample size, sampling methods, and be more specific on outcomes e.g., prevalence of overweight and obesity and its contributing factors among the Indian adolescents.

Before finalization, all the feedback from the investigators will be considered to update the data extractions from the included studies.

### Collating, summarizing, and reporting the results

The aim of the proposed scoping review will be to collect the existing evidence around studies on the prevalence of overweight and obesity among adolescents and the contributing factors; it also aims to summarize the results as reported in the included studies so as to identify gaps in existing research. All these evidence will be proceeded to summarization, analysis and final manuscript preparation with a focus on the outcomes of the studies. The findings from the included studies will be analyzed to identify common themes. The data will be extracted manually to the relevant themes. The themes will be collated, summarized, and reported around the outcomes such as prevalence of overweight and obesity, risk factors etc. Other emerging themes will be reported.

### Ethics and dissemination

This scoping review does not require ethical approval as data will be obtained through review of existing literature. This protocol will provide an exceptional overview of the literature available on the adolescent overweight and obesity in India. The proposed scoping review will systematically map the evidence to identify research priorities and uncover the research gaps around adolescent overweight/obesity issues in India. The potential gaps will provide important inputs to policymakers and various healthcare agencies to outline new research questions and interventions to improve adolescent health in India.

### Patient and public involvement

No patient involved.

## Discussion

The proposed scoping review aims to look for the evidences from various community level studies as well as large-scale national-level studies to analyze and present an overall status on overweight and obesity among adolescents. The prevalence of overweight and obesity among adolescents is a looming threat to public health in India as this nutritional extreme has evolved as a major health issue among adolescents due to their risky eating and dietary behaviors. NFHS-3 data showed, in the age group 15–19 y, 2.4% of girls and 31.7% of boys were overweight [20]. On the other hand, there is an increasing trend of falling physical exercise among adolescents. Particularly, the adverse effect of various forms of media influence is clearly shown in a study from Chennai done in the age group 11 to 17 y reporting that 90% eat either food or snacks while watching TV, 82% buy food products and snacks based on the advertisement, 59% skipped outdoor activities for TV [21]. In this scenario, it is predicted that the obesity prevalence percentage will reach 6.2% by 2030. Improper dietary patterns, unhealthy food habits, and lack of physical activity are the major associated factors that increase the burden of obesity among adolescents [18, 22].

In the above context, this is the first scoping review on the prevalence of obesity and overweight among adolescents in India. We believe this scoping review will provide a wide range of evidence by significantly adding to the already available data and literature resources on adolescent overweight and obesity in India. The present findings regarding adolescent overweight and obesity will represent the national level status on the prevalence rate and pattern of overnutrition among adolescents and identify research gaps that will provide inputs for further research. It will also give insights into nutrition-based policy formulation and implementation.

## Supporting information

S1 File PRISMA-ScR checklist. (DOCX)

S2 File Draft of search strategy to be used using PubMed electronic database. (DOCX)
