# Supplementary material for: The incidence, prevalence, and contributing factors of overweight and obesity among adolescent population of India: A scoping review protocol
Source: PLoS One. 2022 Sep 26;17(9):e0275172. doi: 10.1371/journal.pone.0275172 (PMC9512208; doi:10.1371/journal.pone.0275172)
Supplement: S2 File — (DOCX) [file pone.0275172.s002.docx]

**S2 File. Draft of search strategy to be used using PubMed electronic database**

| **Components** | **Search items** | **Results** |
| --- | --- | --- |
| #1 | **obesity:** "obese"[All Fields] OR "obesity"[MeSH Terms] OR "obesity"[All Fields] OR "obese"[All Fields] OR "obesities"[All Fields] OR "obesity's"[All Fields] | **425,991** |
| #2 | **adolescents:** "adolescences"[All Fields] OR "adolescency"[All Fields] OR "adolescent"[MeSH Terms] OR "adolescent"[All Fields] OR "adolescence"[All Fields] OR "adolescents"[All Fields] OR "adolescent's"[All Fields] | **2,260, 921** |
| #3 | **overweight:** "overweight"[MeSH Terms] OR "overweight"[All Fields] OR "overweighted"[All Fields] OR "overweightness"[All Fields] OR "overweights"[All Fields] | 288,528 |
| #4 | **overnutrition:** "overnutrition"[MeSH Terms] OR "overnutrition"[All Fields] | 259,111 |
| #5 | **Indian:** "Indian"[All Fields] OR "Indian's"[All Fields] OR "Indians"[All Fields] | 31,652 |
| #6 | **Teenager:** "adolescent"[MeSH Terms] OR "adolescent"[All Fields] OR "teenage"[All Fields] OR "teenager"[All Fields] OR "teenagers"[All Fields] OR "teenaged"[All Fields] OR "teenager's"[All Fields] OR "teenages"[All Fields] | 2,220,471 |
| #7 | **Young adults:** "young adult"[MeSH Terms] OR ("young"[All Fields] AND "adult"[All Fields]) OR "young adult"[All Fields] OR ("young"[All Fields] AND "adults"[All Fields]) OR "young adults"[All Fields] | 1,188,667 |
| #8 | **#1 AND #2 AND #3 AND #5**  ("overweight"[MeSH Terms] OR "overweight"[All Fields] OR "overweighted"[All Fields] OR "overweightness"[All Fields] OR "overweights"[All Fields]) AND ("obeses"[All Fields] OR "obesity"[MeSH Terms] OR "obesity"[All Fields] OR "obese"[All Fields] OR "obesities"[All Fields] OR "obesity s"[All Fields]) AND ("adolescences"[All Fields] OR "adolescency"[All Fields] OR "adolescent"[MeSH Terms] OR "adolescent"[All Fields] OR "adolescence"[All Fields] OR "adolescents"[All Fields] OR "adolescent s"[All Fields]) AND ("india"[MeSH Terms] OR "india"[All Fields] OR "india s"[All Fields] OR "indias"[All Fields]) | 762 |
| #9 | **#4 AND #2 AND #5**  ("overnutrition"[MeSH Terms] OR "overnutrition"[All Fields]) AND ("adolescences"[All Fields] OR "adolescency"[All Fields] OR "adolescent"[MeSH Terms] OR "adolescent"[All Fields] OR "adolescence"[All Fields] OR "adolescents"[All Fields] OR "adolescent s"[All Fields]) AND ("india"[MeSH Terms] OR "india"[All Fields] OR "india s"[All Fields] OR "indias"[All Fields]) | 679 |
| #10 | **#4 AND #6 AND #5**  ("overnutrition"[MeSH Terms] OR "overnutrition"[All Fields]) AND ("adolescent"[MeSH Terms] OR "adolescent"[All Fields] OR "teenage"[All Fields] OR "teenager"[All Fields] OR "teenagers"[All Fields] OR "teenaged"[All Fields] OR "teenager s"[All Fields] OR "teenages"[All Fields]) AND ("india"[MeSH Terms] OR "india"[All Fields] OR "india s"[All Fields] OR "indias"[All Fields]) | 660 |
| #11 | **#4 AND #7 AND #5**  ("young adult"[MeSH Terms] OR ("young"[All Fields] AND "adult"[All Fields]) OR "young adult"[All Fields] OR ("young"[All Fields] AND "adults"[All Fields]) OR "young adults"[All Fields]) AND ("overnutrition"[MeSH Terms] OR "overnutrition"[All Fields]) AND ("india"[MeSH Terms] OR "india"[All Fields] OR "india s"[All Fields] OR "indias"[All Fields]) | 430 |
